# Editorial: Interferon response against viral infections

**DOI:** 10.3389/fmicb.2024.1496035

**Published:** 2024-10-11

**Authors:** Ana Maria Ortega-Prieto, Jose Angel Regla-Nava, Jose M. Jimenez-Guardeño

**Affiliations:** ^1^Departamento de Microbiología, Universidad de Málaga, Málaga, Spain; ^2^Instituto de Investigación Biomédica de Málaga y Plataforma en Nanomedicina-IBIMA Plataforma BIONAND, Málaga, Spain; ^3^Department of Microbiology and Pathology, University Center for Health Science (CUCS), University of Guadalajara, Guadalajara, Mexico

**Keywords:** interferon, virology, virus, infectious disease, innate immunity

Viral infections are marked by a constant battle between the invading virus and the infected host. The outcome of this engagement heavily relies on the exploitation of host-encoded factors, which viruses leverage to grow and spread, and the host's immune response, which aims to suppress and eliminate the infection. Among the host defenses, the interferon (IFN) response stands out as a crucial early barrier, mediating the induction of an antiviral state in the cells by leading to the up-regulation of a wide range of IFN-stimulated genes (ISGs) with antiviral properties. On the other hand, to establish a successful infection, many viruses have evolved sophisticated strategies to evade these immune responses by targeting various elements of the innate immune system, thereby allowing them to persist and replicate within the host. In brief, IFNs are essential proteins that, via several mechanisms, regulate cell growth and the inflammatory innate response, and possess immunomodulatory activity and physiological functions. Understanding the molecular mechanisms underlying the innate immune response to viral infections is essential for uncovering points of viral vulnerability, which could guide the development of novel antiviral therapies.

This Research Topic aimed to highlight recent advancements in our understanding of the molecular mechanisms that regulate the host's innate immune response to viral infections. We welcomed contributions across a range of formats, including reviews, original research articles, methods, short communications, case reports, and opinions. In total, the selected articles published in this Research Topic include five research articles and two reviews.

The study by Villamayor et al. exemplifies this focus by exploring the role of IFN alpha-inducible protein 27 (IFI27) in modulating innate immune responses. Their research provides valuable insights into how IFI27 counteracts the innate immune responses triggered by cytoplasmic RNA recognition and binding, thereby influencing the replication of major viruses such as influenza A virus (IAV), severe acute respiratory syndrome coronavirus 2 (SARS-CoV-2), and Sendai virus. Specifically, this research uncovers that IFI27 interacts with the pattern recognition receptor (PRR) retinoic acid-inducible gene I (RIG-I), a cytosolic pattern recognition receptor, impairing its activation. This interaction is likely mediated by IFI27 binding to RNA, which disrupts the antiviral response pathways initiated by cytoplasmic RNA recognition. By dampening these immune signals, IFI27 allows for enhanced viral replication in both *in vitro* and *in vivo* models. While the findings offer important implications in the identification of novel targets for therapeutic intervention to control viral infections, limitations include the need for broader validation across different viral strains and variants and the potential difficulty in targeting IFI27 without impacting other essential cellular processes.

Fu et al. systematically characterized the gene known as the stimulator of IFN genes (STING) of the Brazilian free-tailed bat *Tadarida brasiliensis*, shedding light on its critical role in regulating IFN-β responses during RNA virus infections. This study provides novel insights into why bats are able to act as potential reservoirs for a wide array of highly pathogenic RNA viruses without developing severe disease. Specifically, this work demonstrates that overexpression of BatSTING in lung cells from *T. brasiliensis*, when stimulated by cGAS, significantly reduces RNA virus replication, including vesicular stomatitis virus (VSV). This antiviral effect is closely linked to the ability of BatSTING to regulate the basal expression of IFN-β and different ISGs. Additionally, they described that BatSTING can be activated by a variety of RNA viruses, and its knockdown severely impairs the IFN-β response, confirming its essential function in controlling RNA virus infection. The study represents a key step in understanding viral tolerance in bats. However, the absence of *in vivo* experiments and the limited availability of bat cell models, combined with the diversity of bat species, highlight the need for further investigation into the specific mechanisms of cGAS-STING signaling involved in IFN production against RNA viruses.

Yang et al. used a primary bronchial epithelial cell model to investigate how varying levels of the multifunctional viral protein NS1 influence the host's immune response to IAV infection. This study provides new insights into the hierarchical role of NS1 in targeting different pathways of the host immune response, suggesting that the virus adapts its immune antagonism strategy based on the stage of replication and NS1 concentration. Specifically, they demonstrated that, at single-cell resolution, lower levels of NS1 are sufficient to block immune detection and suppress pro-inflammatory cytokine production, including IFNs. On the other hand, higher concentrations of NS1 are needed to inhibit IFN signaling and the expression of ISGs. Interestingly, the study also uncovered that the highest levels of NS1 are required to inhibit cellular chaperones related to the unfolded protein response (UPR), a process linked to later stages of viral replication. Understanding the precise mechanisms by which NS1 modulates the immune response could reveal novel strategies for antiviral therapies. Additionally, exploring the interactions between NS1 and host cellular pathways may provide insights into how to effectively disrupt viral replication while enhancing the host's antiviral defense.

Wang et al. provide a comprehensive review focused on the multifaceted role of the hepatitis B virus (HBV) non-structural protein HBx in viral replication, modulation of the host immune system, and the development of hepatocarcinogenesis. By manipulating these pathways, HBx enables HBV to evade the host's innate immune response by targeting key signaling components, therefore facilitating persistent infection and contributing to the progression of liver cancer. Interestingly, HBx plays a significant role in disrupting the production of crucial mediators in the antiviral immune response involved in the IFN signaling cascade, such as RIG-I and the interferon regulatory factor 3 (IRF3), effectively inhibiting their function and preventing the activation of antiviral pathways. This interference not only reduces the production of IFN but also impairs downstream signaling, compromising the host's ability to generate an effective immune response against HBV. The review highlights the complex mechanisms by which HBx regulates HBV replication and its critical influence on the IFN signaling pathway, as well as its role in hepatocellular carcinoma metastasis and invasion. While the complexity of HBx's interactions with host immune responses presents significant challenges that must be addressed to translate these insights into effective therapeutic strategies, this understanding provides a crucial foundation for advancing future research and developing therapeutics that specifically target HBx.

In their review, Liu et al. explore the unique role of type III IFN (IFN-λ), a relatively new addition to the IFN family, in antiviral immunity against respiratory viral infections. Unlike type I IFNs, which are widely known for their antiviral functions, IFN-λ operates predominantly at mucosal surfaces, particularly in epithelial cells, and plays a non-redundant role in preventing viral spread. Interestingly, the review highlights the promising therapeutic potential of targeting IFN-λ to enhance respiratory mucosal immunity. However, further clinical trials are essential to validate these findings and fully understand the implications for antiviral therapies.

Zhang B. et al. investigated the isolation of *Limosilactobacillus mucosae* G01, a lactic acid bacterium, from the fecal microbiota of Bama pigs and its antiviral properties against porcine epidemic diarrhea virus (PEDV), a major cause of severe diarrhea in piglets that leads to substantial economic losses in the pig farming industry. Their study revealed that *L. mucosae* G01 exhibits potent inhibitory effects PEDV by enhancing the phosphorylation of IRF3 in IPEC-J2 cells, which in turn induces the production of various IFNs (IFN-α, IFN-β, IFN-λ1 and IFN-λ3). Additionally, the upregulation of ISGs such as MX1, MX2, OAS1, and ZAP was observed in a dose-dependent manner, demonstrating a robust antiviral response and effectively mitigating PEDV replication. This work suggests that *L. mucosae* G01 could serve as a promising and naturally derived alternative for controlling PEDV and potentially other enteric coronavirus infections. However, the scalability and clinical efficacy of such probiotic therapies remain a limitation to be addressed.

Zhang Y. et al. examined the interaction between fungal infections and human papillomavirus (HPV) in the context of cervical lesions. Their research revealed that co-infection with cervical fungi may decrease the risk of cervical lesions, compared to HPV infection alone, suggesting that fungal infection might mitigate the effect of HPV that enhances lesion development. Specifically, the study found that co-infected individuals had a 27% lower risk of developing low-grade squamous intraepithelial lesions (LSIL), a 35% lower risk for high-grade squamous intraepithelial lesions (HSIL), and a 43% reduction in the risk of cervical cancer. Cervical cancer ranks second among women's malignancies, primarily linked to persistent high-risk HPV infection. The immune response, particularly the IFN signaling pathway, plays a crucial role in controlling HPV infections by enhancing the antiviral state of host cells. Interestingly, the review highlights that infections with *Candida albicans* and other fungi can alter host immune responses, potentially influencing HPV persistence. These fungal infections may create an inflammatory environment that disrupts the delicate balance of the immune system, while also enhancing the immune response to HPV vaccines by stimulating T cell proliferation, suggesting a potential adjuvant role. However, several limitations exist in this study, including the challenge of establishing causal relationships between cervical fungal infections and decreased HPV persistence or cervical lesion incidence due to its cross-sectional design and the lack of detailed insights into the underlying molecular mechanisms. Future longitudinal studies, along with investigations into the molecular pathways involved, are essential to further elucidate these complex interactions and their implications for cervical cancer prevention.

Together, the articles presented in this Research Topic provide valuable advancements in our understanding of the complex interaction between viral infections and host immune responses, with a particular emphasis on the role of the IFN response. From the identification of mechanisms by which viruses, like HBV and IAV, evade the immune response to the exploration of potential therapeutic interventions such as probiotics, these studies pave the way for future research that could translate into innovative antiviral therapies. For instance, the investigation into the impact of IFI27 on innate immune responses reveals how specific cellular proteins can affect viral infections. Similarly, the exploration of *L. mucosae* G01 demonstrates how certain beneficial microbes can enhance IFN-mediated antiviral responses. By focusing on the molecular dynamics of immune modulation and viral survival strategies, these findings hold the potential to inspire new approaches in both antiviral drug development and immune-based interventions, ultimately contributing to improved disease control and prevention strategies.

